# Supplementary material for: Spatial Variation in Stream-Water Bacterial Communities Across Sites Differing in Wild Chinese Giant Salamander Detection Frequency
Source: Biology (Basel). 2026 Sep 18;15(18):1647. doi: 10.3390/biology15181647 (PMC13604818; doi:10.3390/biology15181647)
Supplement: Supplementary file 1 [file biology-15-01647-s001.zip › Data Related to “Spatial Variation in Stream-Water Bacterial Communities across Sites Differing in Wild Chinese Giant Salamander Detection Frequency”/Microbial Data/report/report S1.html]

诺禾致源16S扩增子QIIME2分析报告


|  |  |  |  |  |  |  |
| --- | --- | --- | --- | --- | --- | --- |
|  |  |  |  |  |  |  |


# 诺禾致源16S扩增子QIIME2分析报告

|  |  |
| --- | --- |
| 合同编号 | X101SC25121510-Z01 |
| 合同名称 | 果茶所60个特异性引物扩增子测序分析技术服务（委托）合同 |
| 分期编号 | X101SC25121510-Z01-J007 |
| 扩增区域 | 16SV34 |
| 引物序列 | CCTAYGGGRBGCASCAG,GGACTACNNGGGTATCTAAT |
| 引物名称 | 341F,806R |
| 报告时间 | 2026-04-02 |
| 项目概述 | 总计6个样本，测序共得到双端reads（RawPE）629851.0条，拼接得到tags（Tags）629724.0条，得到ASV/OTU 5738个。 每个样本的reads数量从78794.0条到97235.0条,为保证样本间对比所使用的测序深度一致，每个样本选取69135条reads进行后续分析，保证测序深度相同。 |
| 售后服务电话 | 400-658-1585 |
| 售后服务邮箱 | service-meta@novogene.com |
| 售后服务云平台 | https://magic-plus.novogene.com/  说明：收到数据释放邮件的下一个工作日，您将收到售后工具平台项目权限开通的邮件;届时您可以根据邮件提示登录云平台并进行数据分析； |
| 温馨提示 | Report仅供展示部分结果，全部分析内容详见Result文件夹。 在您拿到Result前Report中的Result超链接无效，确认结算后，释放结果文件Result中Report的结果目录超链接有效。 如对结题报告展示或结果文件位置存在疑问，请阅读ReadMe.html，若未能解答您的疑问，请电话或邮件联系售后服务组，我们竭诚为您服务。 |
